# An RNA-Seq Transcriptome Analysis of Histone Modifiers and RNA Silencing Genes in Soybean during Floral Initiation Process

**DOI:** 10.1371/journal.pone.0077502

**Published:** 2013-10-16

**Authors:** Lim Chee Liew, Mohan B. Singh, Prem L. Bhalla

**Affiliations:** Plant Molecular Biology and Biotechnology Laboratory, ARC Centre of Excellence for Integrative Legume Research, Melbourne School of Land and Environment, the University of Melbourne, Parkville, Victoria, Australia; Harbin Institute of Technology, China

## Abstract

Epigenetics has been recognised to play vital roles in many plant developmental processes, including floral initiation through the epigenetic regulation of gene expression. The histone modifying proteins that mediate these modifications involve the SET domain-containing histone methyltransferases, JmjC domain-containing demethylase, acetylases and deacetylases. In addition, RNA interference (RNAi)-associated genes are also involved in epigenetic regulation via RNA-directed DNA methylation and post-transcriptional gene silencing. Soybean, a major crop legume, requires a short day to induce flowering. How histone modifications regulate the plant response to external cues that initiate flowering is still largely unknown. Here, we used RNA-seq to address the dynamics of transcripts that are potentially involved in the epigenetic programming and RNAi mediated gene silencing during the floral initiation of soybean. Soybean is a paleopolyploid that has been subjected to at least two rounds of whole genome duplication events. We report that the expanded genomic repertoire of histone modifiers and RNA silencing genes in soybean includes 14 histone acetyltransferases, 24 histone deacetylases, 47 histone methyltransferases, 15 protein arginine methyltransferases, 24 JmjC domain-containing demethylases and 47 RNAi-associated genes. To investigate the role of these histone modifiers and RNA silencing genes during floral initiation, we compared the transcriptional dynamics of the leaf and shoot apical meristem at different time points after a short-day treatment. Our data reveal that the extensive activation of genes that are usually involved in the epigenetic programming and RNAi gene silencing in the soybean shoot apical meristem are reprogrammed for floral development following an exposure to inductive conditions.

## Introduction

Flowering is a crucial process during the life cycle of plants which determines reproductive success in plant and underpins productivity in agriculture. Grains legumes including soybeans (*Glycine max*) are second only to cereal crops as a source of food and feed. Soybean is one of the world’s most important crops, particularly for most of the worldwide oilseed production. In term of flowering characteristics, soybean is a short-day plant, nonvernalisation-responsive species, and flower reversion can be induced in soybean when plants are transferred from long-day to short-day condition. In addition, soybean cultivars from different maturity groups display variability in the photoperiod (and/or temperature) stimulus requirements for the initiation of flowering [1]. Recent studies on the underlying mechanisms for floral initiation process in soybeans and other legumes species has highlighted the unique aspect on legume reproduction [2–6]. 

In the model plant, *Arabidopsis thaliana*, genetic pathways for flowering regulation have been well characterised which involve four major pathways – photoperiod, vernalisation, autonomous, and hormones in order to coordinate with exogenous environmental cues (light and temperature) and endogenous developmental cues (ages and developmental stages) [7,8]. Based on our current knowledge on flowering, exogenous signals is perceived by plants through receptors in the leaf, where a mobile floral stimulus (“florigen”) is produced and translocated from the leaves to the shoot apex to triggers flowering by inducing the expression of floral meristem identity genes in the shoot apical meristem [9,10]. Recently in plant, epigenetics has shown to be involved in the control of flowering and also other plant growth and development stage including embryogenesis, organogenesis and stress response [11–14]. 

In eukaryotic cells, the genetic information is stored in chromatin which is organised in nucleosome units composed of histone proteins that are wrapped by highly compacted DNA. In addition to regulating gene expression at the transcriptional and post- transcriptional levels, the degree of chromatin compaction governs the accessibility of transcriptional machinery proteins and hence the activation of genes. “Epigenetics” is an extra level of control on gene expression without changes on DNA sequence, mechanisms involved including histone modification, DNA methylation, RNA-mediated chromatin silencing. Repeating nucleosome units in chromatin consist of an octamer of histone H2A, H2B, H3 and H4 protein dimers. Histone modifications primarily occur at the N-terminal of histone tails which include covalent modification such as acetylation, methylation, phosphorylation, and ubiquitylation. Histone modifications such as acetylation have been associated with euchromatin formation (loosely packed chromatin structure) and transcriptional activation whereas others such as methylation lead to tightly packed chromatin (heterochromatin) and inactive transcription. Different histone modifiers (or histone modifying enzymes) are known to carry out these histone modification processes and they are highly conserved in human, animals, yeast and plant [15–18]. 

**Figure 1:**
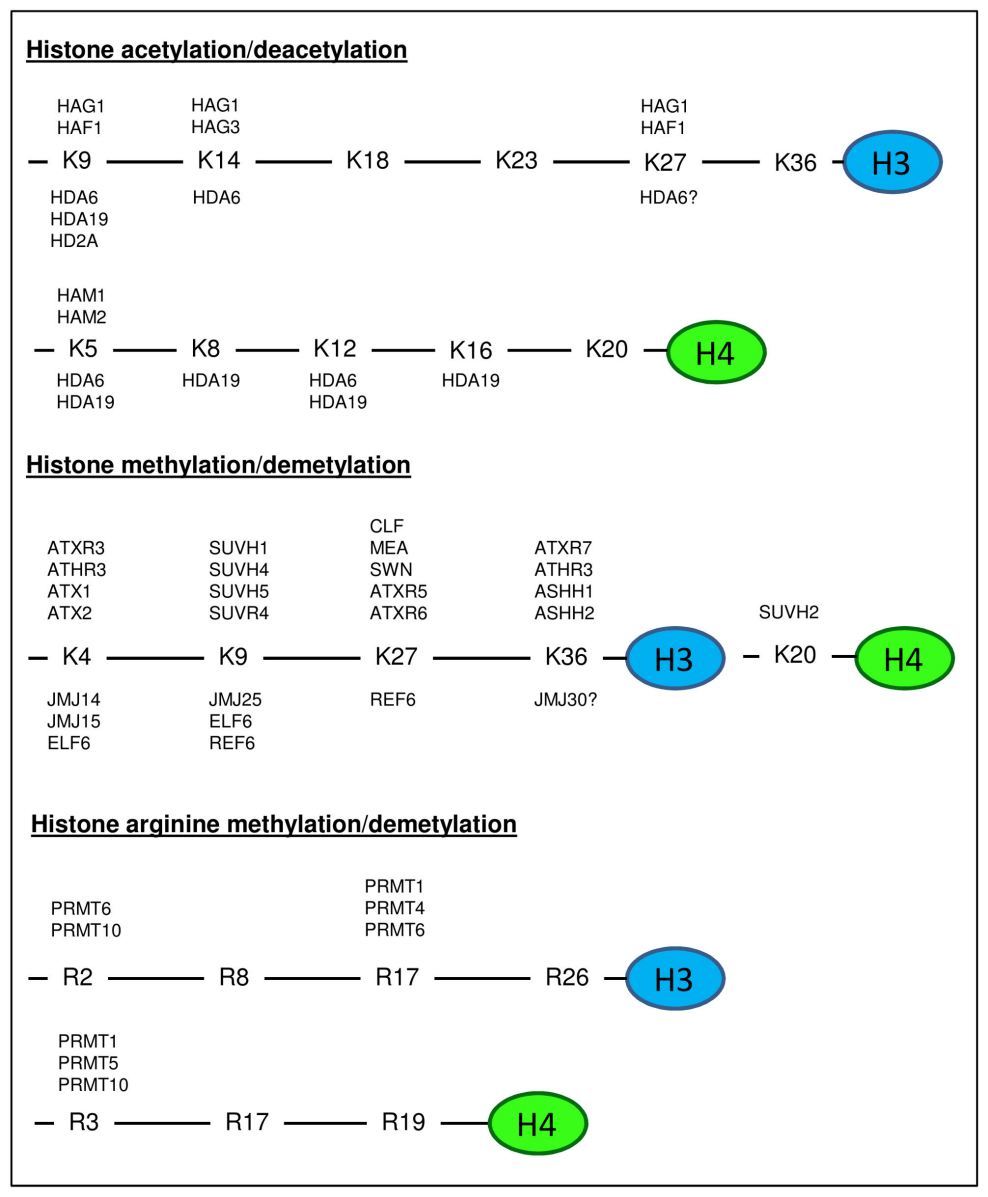
Plant histone modifiers and their site of modification on the H3 and H4 histone tails. The figure represents the histone modifications on specific residues of H3 and H4 histone tails with the different histone modifiers. Several of the basic amino acids – Lysine (K), Arginine (R) are targeted for histone acetylation/deacetylation, histone lysine methylation/demethylation and arginine methylation/demethylation by numerous histone modifying enzymes –histone acetyltransferase (HAG, HAF, HAM ), histone deacetylase (HDA), histone methyltransferase (ATX, ATXR, ATHR, SUVH, SUVR, CLF, MEA, SWN, ASHH), Jumonji demethylase (JMJ), Early flowering 6 (ELF6), Relative of ELF6 (REF6), protein arginine methyltransferase (PRMT).

Histone acetylation is a dynamic and reversible process. Histone acetylation and deacetylation is addition or removal of acetyl group on the lysine (K) residue of histone H3 and H4, which is carried out by histone acetyltransferase (HATs) and histone deacetylase (HDACs) respectively. The targets of HATs and HDACs on H3 tail are lysine residue 9, 14, 18, 23, 27 and 36, and on H4 tail are lysine residue 5, 8, 12, 16, and 20 [19,20]. HATs and HDACs are highly conserved in human, animals, yeast and plant, and can be classified into different groups based on their protein domain composition. HATs are classified into (1) CBP (CREB-BINDING PROTEIN) (2) GNAT (GCN5-RELATED N-ACETYLTRANSFERASES)/MYST (Moz, Ybf2/Sas3, Sas2, Tip60) (3) TAF_II_250 (TBP-ASSOCIATED FACTOR) class [20,21]. In *Arabidopsis*, specific HATs or HDACs are known to acetylate or deacetylate certain lysine residues (Figure 1) such as members from GNAT/MYST class - AtHAG1/AtGCN5 acetylates H3K9, H3K14, and H3K27, AtHAM1/2 acetylates H4K5, and AtHAG3 acetylates H3K14. On the other hand, HDACs include (1) HD2 (HISTONE DEACETYLASE 2), (2) SIR2 (SILENT INFORMATION REGULATOR PROTEIN 2), and (3) HDAs (HISTONE DEACETYLASE) class (Class I – III) [22–24]. Amongst them, AtHDA6 and AtHDA19/HD1 from the HDAs class I are the best-studied HDACs which are shown to be involved in removal of a few of the acetylation marks on H3 and H4 tails [25–27]. The HD2 class of HDACs is specific to plants and not found in other kingdom, AtHD2A was found to work as a H3K9 deacetylase [28,29].

Histone methylation is the addition of one (me), two (me2) or three methyl (me3) groups to the amino-terminal tails of histone H3 and H4, and mainly occurs at lysine residues of 4, 9, 27, and 36 of H3, and 20 of H4. These covalent modifications are carried out by the histone modifiers – histone methyltransferase (HMTs) and histone demethylases (HDMs). In plants, all the known HMTs have a well-conserved SET (Su(var)3-9, Enhancer-of-zeste, Trithorax) domain and therefore also named as SET domain groups (SDG) proteins [30]. In *Arabidopsis*, HMTs can be categorised into five classes – Class I to V [31,32]. *Arabidopsis* E(z) proteins, MEA (MEDEA), CLF (CURLY LEAF) and SWN (SWINGER) are class I SDG proteins, like the Drosophila and mammalian E(z) proteins, they catalyse H3K27me3 which is associated with gene repression [33]. Class II SDG proteins are ASH1 (ABSENT OF SMALL HOMEOTIC DISCS 1) proteins (e.g. ASHH1, ASHH2, ATHR3) methylate both H3K4me3 and H3K36 (Figure 1) [34]. TRX (TRITHORAX) proteins (e.g. ATX1, ATX2, ATXR3, ATXR7) belong to class III SDG class and are H3K4me3 methyltransferases [35,36]. ATXR5 and ATXR6 (ATX1-RELATED 5 and 6) are class IV SDGs which function to add single methyl group to H3K27 [37]. *Arabidopsis* class V SDG, SU(VAR)3-9 members (e.g. SUVH1, SUVH4, SUVH5, SUVR4) are responsible for H3K9 methylation which are correlated to heterochromatin formation [38]. Histone methylation can also occur at arginine (R) residue of histone tails and histone modifiers involved are known as protein arginine methyltransferase (PRMTs). A few of them have been characterised in *Arabidopsis* and shown to methylate arginine residue of 2, 17 of H3 and R3 of H4 (Figure 1) [39]. Histone methylation was thought to be irreversible until the discovery of lysine-specific demethylase (LSD1) and the JmjC (Jumonji C) domain containing proteins. There are four LSD1-like genes in *Arabidopsis*, they were showed to be H3K4 mono- and di-demethylase and responsible for epigenetic regulation of the expression level of one of the key floral repressor gene, FLOWERING LOCUS C (FLC) [40,41]. Unlike LDS1 proteins, JmjC proteins are known to demethylate all of the mono-, di- and trimethylated lysines of histones [42]. There are five class in the JmjC-containing proteins – (1) KDM5/JARID (2), KDM3/ JHDM2 (3), KDM4/ JHDM3 (4), JMJD6, and (5) Cupid domain only [43]. About 21 JmjC proteins have been discovered in *Arabidopsis* which are able to demethylate lysine H3K4, H3K9, H3K27, and H3K36 (Figure 1). However, no JmjC protein in plants has shown arginine demethylase activity which has been found in animals [44]. 

Besides the transcriptional gene silencing by histone modification, non-coding RNAs (ncRNAs) also contribute to epigenetic regulation via post-transcriptional gene silencing (PTGS) or RNA-directed DNA methylation (RdDM) [45,46]. The RdDM mechanism involves small interfering RNAs (siRNAs) biogenesis and RNA-induced transcriptional silencing complex which trigger RNA interference (RNAi) activity and DNA methylation [47,48]. In detail, it involves RNAs transcribed by RNA polymerase IV (NRPD1a and NRPD2a) to ssRNA and RNA-DEPENDENT RNA POLYMERASE 2 (RDR2) synthesise the dsRNA which is processed by DICER-LIKE (DCL) and HEN1 to 24-nt siRNAs. The siRNAs are incorporated into ARGONAUTE 4 (AGO4) and together with RNA polymerase V (NRPD1b and NRPD2a) are directed to cytosine methylation by DOMAINS REARRAGED DNA METHYLATION 2 (DRM2). The cytosine methylations are maintained by METHYLTRANSFERASE 1 (MET1) and CHROMOMETHYLASE 3 (CMT3) while can be removed by DNA glycosylase-lyase proteins – REPRESSOR OF SILENCING 1 (ROS1) and DEMETER (DME).

**Figure 2:**
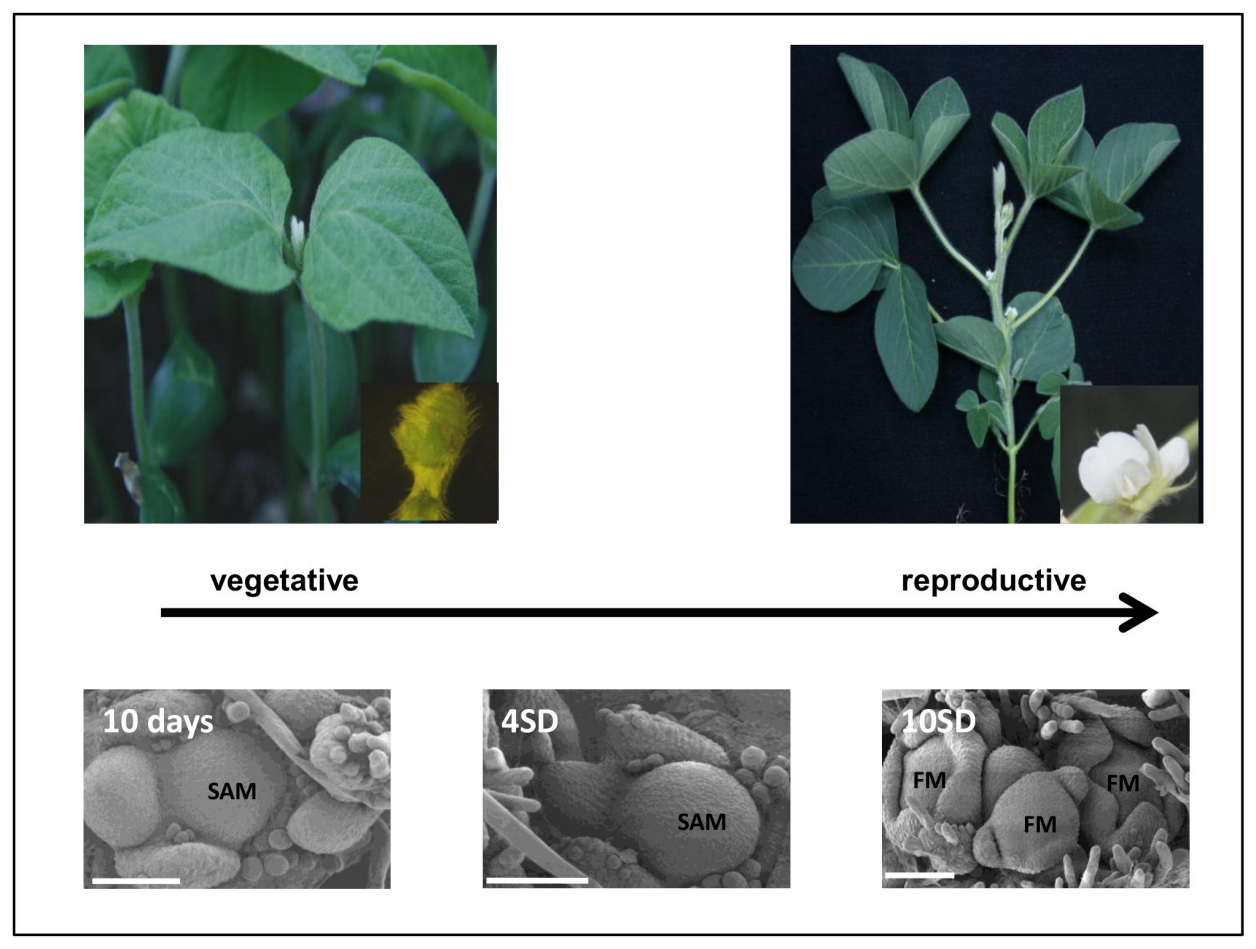
Soybean shoot apical meristem development from vegetative to reproductive stage. Micrograph of scanning electron microscopy of soybean shoot apical meristem from plants of 10 days old, after 4 and 10 short-days (SD) respectively. Shoot apical meristem (SAM), Floral meristem (FM). Micrograph were adapted with permission of John Wiley and Sons from[53].

The epigenetic regulation of gene expression is an important mechanism in the autonomous and vernalisation pathways of flowering control where FLOWERING LOCUS C (FLC), a flowering repressor gene is regulated by epigenetic modification in response to winter’s cold [40,49,50]. However, no evidence has linked epigenetics with photoperiodic flowering. Here, we are interested to investigate involvement of epigenetics during floral initiation in soybean after inductive short-day treatment, particularly at the development from shoot apical meristem to floral meristem (Figure 2). Using the complete and well-annotated genome sequence of soybean [51] as well as transcriptome data in the leaf and shoot apical meristem of soybean after exposure to an inductive short-day treatment [52], we provide a comprehensive overview of the histone modifiers and RNA silencing genes involved in the flower initiation of soybean. In this study, the soybean histone modifier and RNA silencing genes that contribute to the floral initiation process were identified, and the characters of protein structure, phylogeny, and gene expression were examined through bioinformatics analyses. According to our data, the photoperiod-induced reprogramming of the soybean shoot apical meristem to floral development is accompanied by the extensive activation of genes involved in epigenetic programming and RNAi-mediated gene silencing.

## Materials and Methods

### Identification of soybean histone modifiers and RNA silencing genes

The protein sequences of *Arabidopsis thaliana* histone modifiers and RNA silencing components were retrieved from ChromDB (http://www.chromdb.org/) and NCBI (https://www.ncbi.nlm.nih.gov/). The homologous soybean genes were extracted from the transcriptome data of *Glycine max* [52,53] which were captured by RNA-seq libraries from a total of nine samples of leaves and shoot apical meristems (SAMs) from 10 days old soybean seedlings after subjected to 0-4 short-day treatment respectively. The raw data of soybean histone modifiers and RNA silencing transcripts can be found in Table S1. The transcriptome data has been previously deposited in NCBI Sequence Read Archive, under study accession number SRP020868 (run accession number SRR824155-SRR824163) [52]. 

### Protein domains prediction

To determine the specific protein domains of histone modifying enzymes and RNA silencing components, *Arabidopsis thaliana* and *Glyine Max* protein sequences were batch downloaded from The Arabidopsis Information Resource (TAIR) (http://www.arabidopsis.org/tools/bulk/sequences/) and SoyBase, the USDA-ARS Soybean Genetics and Genomics Database (http://www.soybase.org/dlpages/index.php). The protein sequences of all histone modifiers and RNA silencing proteins were analysed for recognizable domains using NCBI Batch conserved domain search tool (http://www.ncbi.nlm.nih.gov/Structure/bwrpsb/bwrpsb.cgi). Domains were also verified using the HMMERbased SMART website (http://smart.embl-heidelberg.de) and PFAM website (http://pfam.sanger.ac.uk/). Schematic diagram of protein domain structures with functional domains were constructed using DOG 2.0 (Domain Graph, version 2.0) (http://dog.biocuckoo.org/) [54].

### Protein alignment and phylogenetic analysis

Protein sequences of *Arabidopsis thaliana* and *Glyine max* retrieved earlier were aligned in the multiple sequences alignment tool CLUSTALX2.0, using Gonnet Protein Weight Matrix with default parameters. Phylogenetic tree was constructed by the Neighbour-Joining algorithm using the MEGA 5.0. The bootstrap consensus tree was inferred from 1000 replicates.

### Transcript abundance analyses and visualization

The expression data of *Glycine max* histone modifier and RNA silencing genes were extracted from transcriptome dataset SRP020868. The abundance for each gene was calculated and expressed in RPKM (reads per kilobase per million reads) in previous studies [52]. Transcripts expressed below 2 RPKM reads in all samples were excluded from further analysis. Heat maps were generated to represent the self-normalised gene expression ratio in percentage with the RPKM value shown in the most highly expressed sample. To visualise the gene expression clusters among homologues and/or paralogs, heat maps were plotted against the phylogenetic tree constructed using soybean protein sequences. K-means clustering was performed with Person correlation using MultiExperiment Viewer (MeV v4.8) with number of clusters = 10 and number of iterations = 50 [55].

## Results and Discussion

To capture the dynamics of transcripts changes in the leaf and SAM as a result of short-day treatment in soybean, transcriptome sequencing was previously performed [52]. As described in our early study [52], transcriptome data were subjected to mapping and annotation analyses against the soybean genome database at Phytozome (www.phytozome.net). The transcript level was calculated and expressed in RPKM. When a soybean histone modifiers or RNAi silencing gene transcript exhibited a significant difference in read abundance in at least one time point relative to the previous time point in either leaf (L1-L0, L2-L1, L3-L2) or SAM (S1-S0, S2-S1, S3-S2, S4- S3), the gene is considered differentially expressed as described by Wong et al. (2013). These genes were extracted from the transcriptome data and subjected to protein domain prediction, phylogenetic and transcript abundance analysis as described in the Materials and methods section. In this study, we have successfully identified 124 histone modifiers and 47 RNAi silencing genes whose transcripts were differentially expressed in the soybean leaf and shoot apical meristem during the flowering initiation process. To further characterise these soybean genes, phylogenetic trees and heat maps were constructed to analyse their evolutionary relationship and expression profiles.

### Histone acetylation/deacetylation

**Figure 3:**
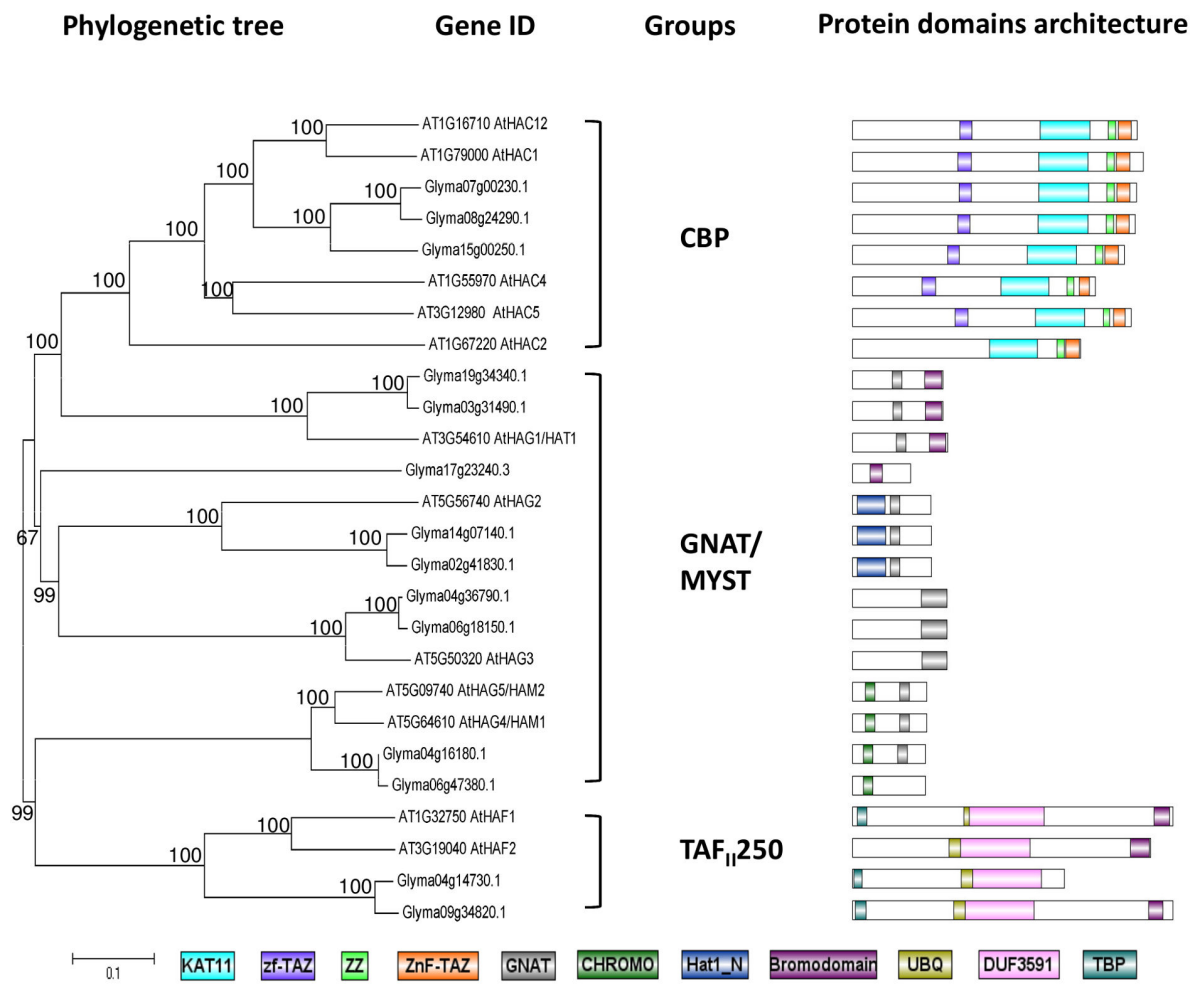
Phylogenetic tree and domain architecture of histone acetyltransferase (HATs). Neighbour-Joining phylogenetic tree are constructed based on protein alignments of *Arabidopsis* and soybean HATs proteins using ClustalX2. Bootstrap values greater than 50% are shown at the nodes and tree is drawn to given scale of branch lengths. The schematic diagrams show the domain organization of these proteins according to analysis by NCBI Batch-CD, SMART and PFAM searches. Different domains are indicated by the use of different colours as shown at the bottom of the figure. The accession numbers for the sequences used in the alignment are listed in the figure.

**Figure 4:**
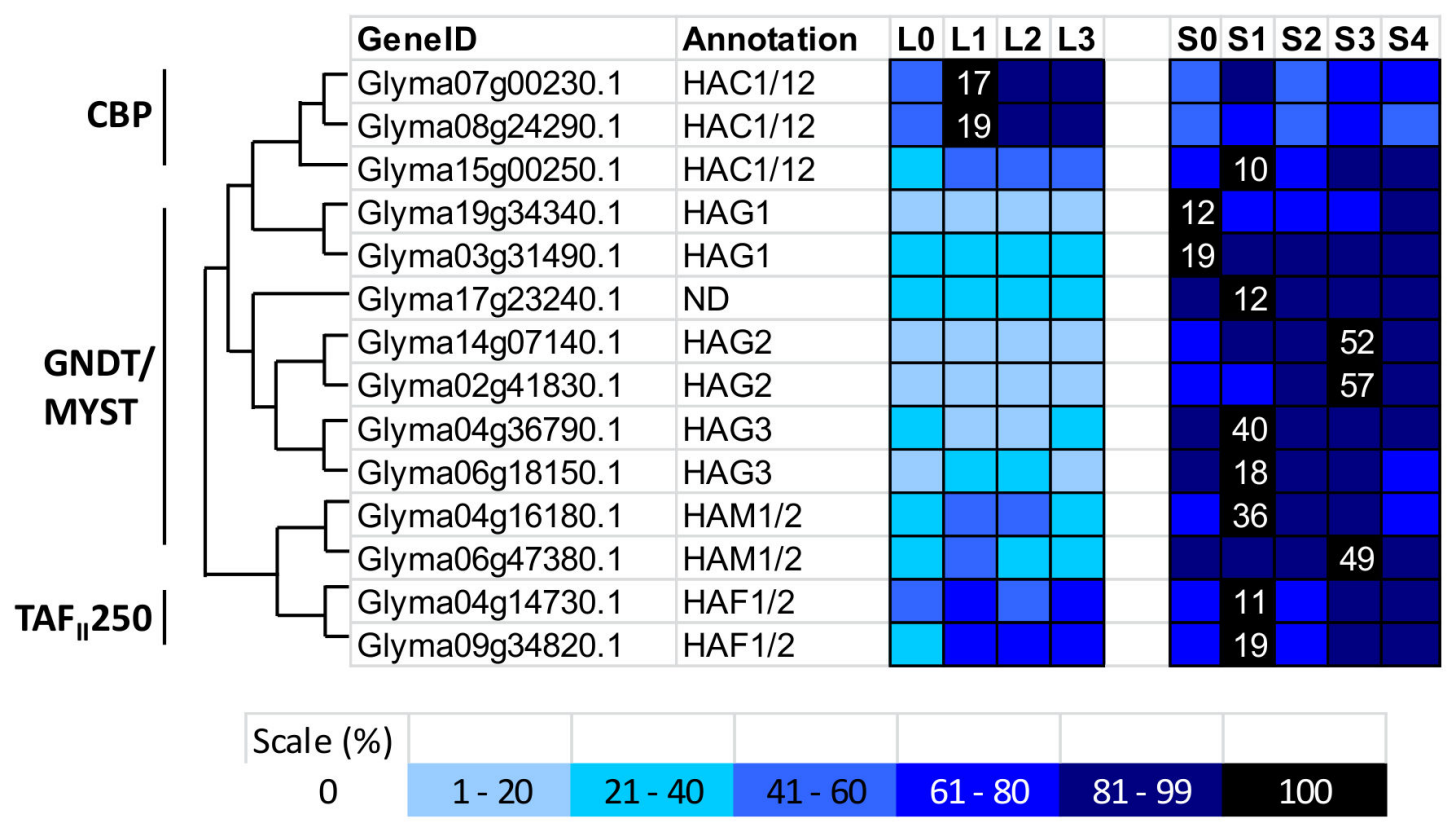
Expression profile and evolutional pattern of histone acetyltransferase (HATs). The heat map shows the relative abundances of soybean genes identified. The highest expression level for each gene across different samples is given in RPKM value. The level of expression for a gene across different samples are represented as percentage of the maximum expression level in colour code from 0% (white) to 100% (black). Heat maps were plotted against the phylogenetic tree constructed using soybean protein sequences and GO annotation. L0, L1, L2, and L3 were leaves at 0 short-day, 1 short-day, 2 short-day, and 3 short-day; S0, S1, S2, S3, and S4 were shoot apical meristems at 0 short-day, 1 short-day, 2 short-day, 3 short-day and 4 short-day.

**Figure 5:**
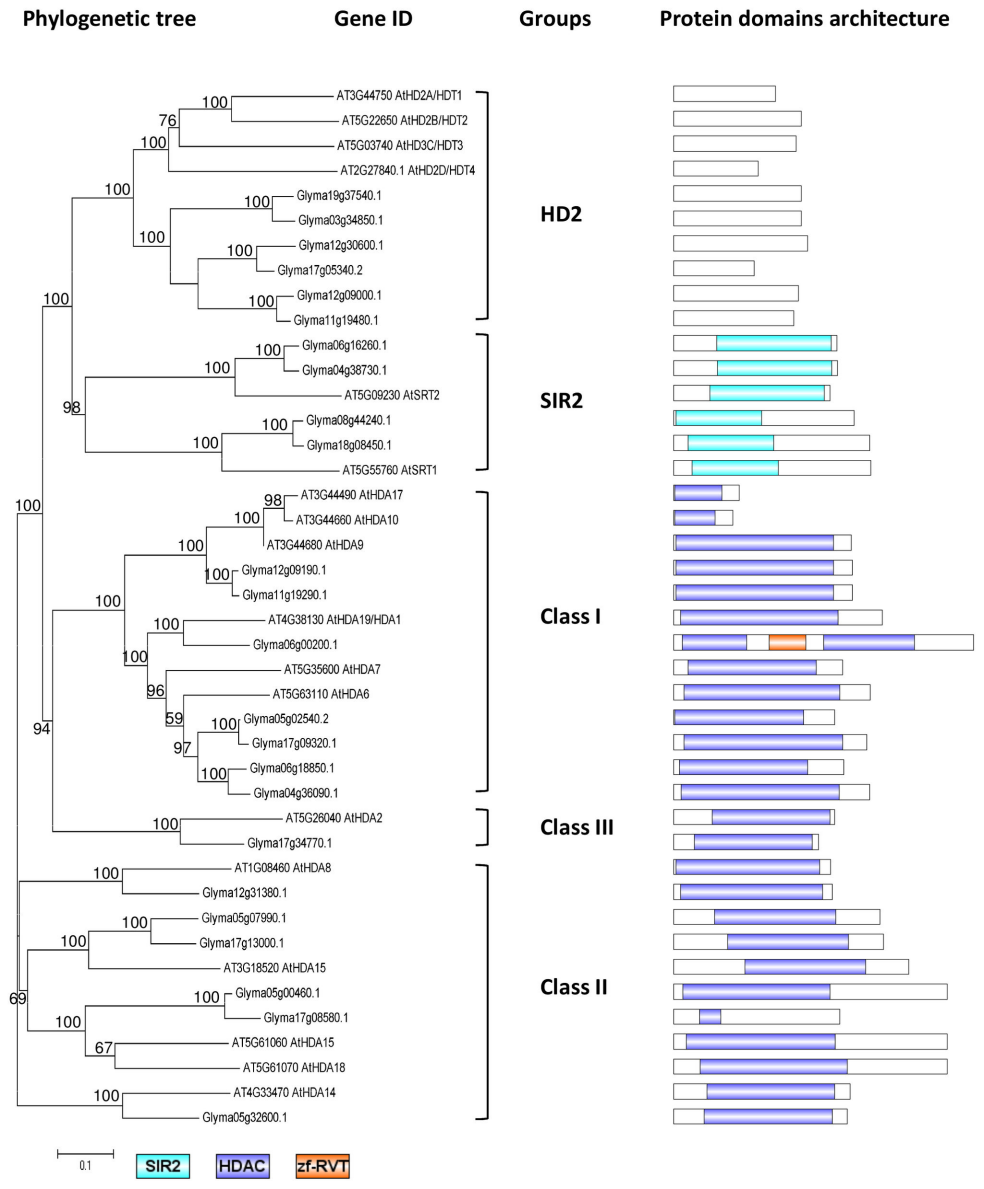
Phylogenetic tree and domain architecture of histone deacetylase (HDACs). Neighbour-Joining phylogenetic tree are constructed based on protein alignments of *Arabidopsis* and soybean HATs proteins using ClustalX2. Bootstrap values greater than 50% are shown at the nodes and tree is drawn to given scale of branch lengths. The schematic diagrams show the domain organization of these proteins according to analysis by NCBI Batch-CD, SMART and PFAM searches. Different domains are indicated by the use of different colours as shown at the bottom of the figure. The accession numbers for the sequences used in the alignment are listed in the figure.

**Figure 6:**
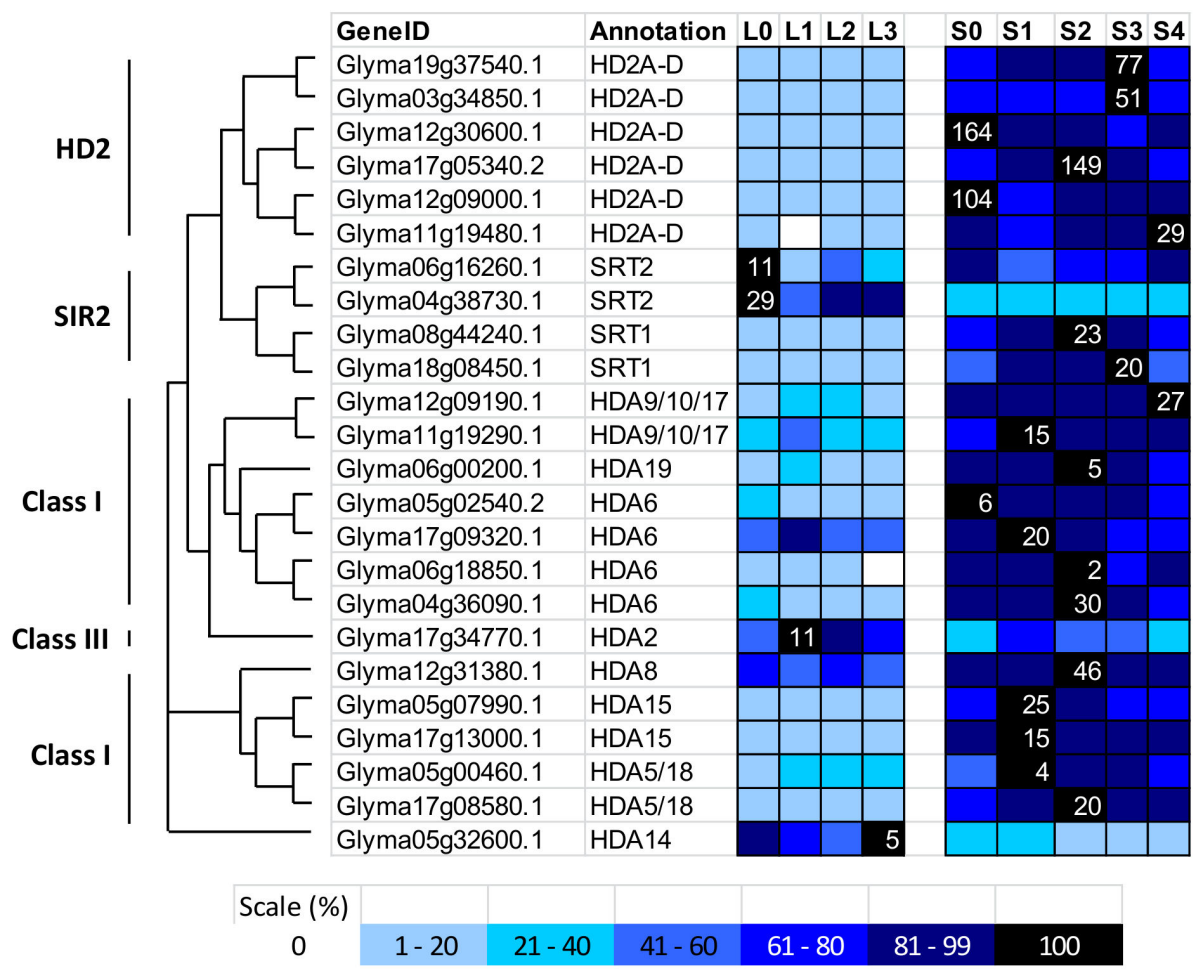
Expression profile and evolutional pattern of histone deacetylase (HDACs). The heat map shows the relative abundances of soybean genes identified. The highest expression level for each gene across different samples is given in RPKM value. The level of expression for a gene across different samples are represented as percentage of the maximum expression level in colour code from 0% (white) to 100% (black). Heat maps were plotted against the phylogenetic tree constructed using soybean protein sequences and GO annotation. L0, L1, L2, and L3 were leaves at 0 short-day, 1 short-day, 2 short-day, and 3 short-day; S0, S1, S2, S3, and S4 were shoot apical meristems at 0 short-day, 1 short-day, 2 short-day, 3 short-day and 4 short-day.

 The *Arabidopsis* genome contains at least 12 HATs and 18 HDACs. Soybean, a well-documented paleopolyploid, has been subjected to at least two rounds of whole genome duplication events and several segmental duplication and genomic rearrangements [56]. Our data demonstrate that soybean genome encodes at least 14 HATs and 24 HDACs whose transcripts are differentially expressed during flower initiation process (Figure 3-6). 

#### Soybean HATs

 There are three classes of plant histone HATs: CBP, GNAT/MYST, and TAF_II_250. The CBP groups contain characteristic protein domains, including the KAT11, zf-TAX, ZZ, and ZnF-TAZ domains. The present survey identified three proteins belonging to this CBP group that cluster with the five Arabidopsis CBP group members (AtHAC1, 2, 4, 5, and 12) [57]. These three proteins share a high protein domains composition with their *Arabidopsis* counterparts, especially AtHAC1 and AtHAC12 while no distinct homologues were found for AtHAC2, AtHAC4, and AtHAC5 in the phylogenetic analysis (Figure 3). In terms of expression profile following an inductive short-day treatment, the putative paralogues of Glyma07g00230.1 and Glyma08g24290.1 show the highest expression levels in leaf after 1 day of induction (Figure 4). Glyma15g00250.1 shows higher expression value in the SAM than in the leaf.

 The second class of HATs is GNAT/MYST class. There are 9 soybean proteins in this class, according to their phylogenetic tree. All of the proteins share domains typical to their Arabidopsis homologues, including a conserved GNAT domain and CHROMO (Chromatin Organisation Modifier, found in proteins involved in the assembly of protein complexes on chromatin), Hat1_N (N-terminal half of histone acetyltransferase HAT1) and bromodomain (recognises acetylated lysine residues on histone tails) [20]. Two soybean homologues were found for each of AtHAG1, AtHAG2, and AtHAG3, and two soybean proteins were found that were highly similar to both AtHAM1 and AtHAM2 (Figure 3). Glyma17g23240.1 contains only the bromodomain was also found to be clustered in GNAT/MYST class. In the transcriptome analysis, all of the soybean members of the GNAT/MYST class showed a higher transcript level in the SAM than in the leaf (Figure 4). The soybean homologues of *AtHAG1* showed highest expression at 0 short day, soybean homologues of *AtHAG2* peaked at 3 short days, and soybean homologues of *AtHAG3* peaked at 1 short day (Figure 4). 

 There are two members, AtHAF1 and AtHAF2, in the *Arabidopsis* TAF_II_250 HATs class. The soybean transcriptome showed 2 TAF_II_250 proteins which share the same protein domain composition as their *Arabidopsis* homologues (Figure 3). These two proteins showed the highest transcript level in the SAM after 1 short day (Figure 4). In *Arabidopsis*, AtHAG1 and AtHAF1 both regulate the light-regulated gene expression via histone acetylation [58].

#### Soybean HDACs

This study revealed 24 HDACs in soybean, and the phylogenetic analysis classified 6 of them in the HD2 class, 4 in the SIR2 class, and 14 in the HDA class (Figure 5). In the plant-specific HD2 class, no known protein domain has been detected [59]. There are six HD2 transcripts detected during soybean flower initiation. In *Arabidopsis*, HD2 deacetylases are associated with abscisic acid (ABA) and the abiotic stress response, and the AtHD2 members’ expression levels were repressed by high levels of ABA and salt [27]. AtHD2C also interacts with other HDACs, including AtHDA6, to regulate gene expression in response to the ABA levels [27]. Our previous studies have reported an increased in the ABA level in the SAM during flowering initiation [52]. In the current study, all 6 soybean members of the HD2 class show high expression levels in the SAM; Glyma12g30600.1 and Glyma12g09000.1 both showed extremely high transcript levels before the short-day treatment, which gradually reduced as the treatment increased (Figure 6). These results are inversely correlated with ABA biosynthetic gene expression [52]. 

 Soybean contains 4 members in the SIR2 histone deacetylase class, 2 of which cluster with AtSRT1 and 2 of which form a separate clade with AtSRT2 (Figure 5). All of the soybean SIR2 proteins contain the distinctive SIR2 domain. The expression profile of the soybean *SRT1* and *SRT2* homologues are remarkably different, as *GmSRT1* is highly expressed in the SAM, while *GmSRT2* is highly expressed in the leaf, suggesting different functions (Figure 6). However, the functions of the SIR2 proteins are currently unclear, even in *Arabidopsis*. In yeast, SIR2 was found to be an NAD (nicotinamide adenine dinucleotide)-dependent histone deacetylase where NAD serve as a co-factor for histone deacetylation activity [60]. 

 The remaining HDACs belong to HDA class, which can be further classified into Class I, II, and III. Based on sequence homology, AtHDA6, AtHDA7, AtHDA9, AtHDA10, AtHDA17, and AtHDA19 belong to Class I, AtHDA5, AtHDA8, AtHDA14, AtHDA15, AtHDA18 belong to Class II, AtHDA2 belong to Class III. A total of 14 soybeans proteins were found to be grouped in the HDA class, 7 in Class I, 6 in Class II, 1 in Class III, and all of these proteins have a highly conserved histone deacetylase (HDAC) domain (Figure 5). Glyma12g31380.1 is the soybean homolog of AtHDA8 and it transcript is expressed in both leaf and SAM at the similar level. Glyma05g32600.1 (*AtHDA14* homologue) and Glyma17g34770.1 (*AtHDA2* homologue) are the only two HDA members have higher transcripts in leaf than SAM. AtHDA6 has highest number of homologues in soybean (Figure 6). Coincidently, it is one of the two histone deacetylases which are best-studied HDAC in plants, showing deacetylase activity at various lysine residues at H3 and H4 (Figure 1) [25,26,61]. There is one soybean homologue for AtHDA19, and it has a zf-RVT domain spanning the HDAC domains (Figure 5). The zf-RVT domain is normally found in reverse transcriptase, and this is the first combination of these domains reported to date. 

### Histone methylation/demethylation

There are at least 31 SET domain histone methyltransferases, 9 protein arginine methyltransferase and 21 JmjC-containing demethylases genes present in *Arabidopsis* genome. In this study, we have successfully identified 47 SET domain group (SDG) proteins, 15 PRMTs and 24 JmjC proteins.

#### Soybean SDGs

**Figure 7:**
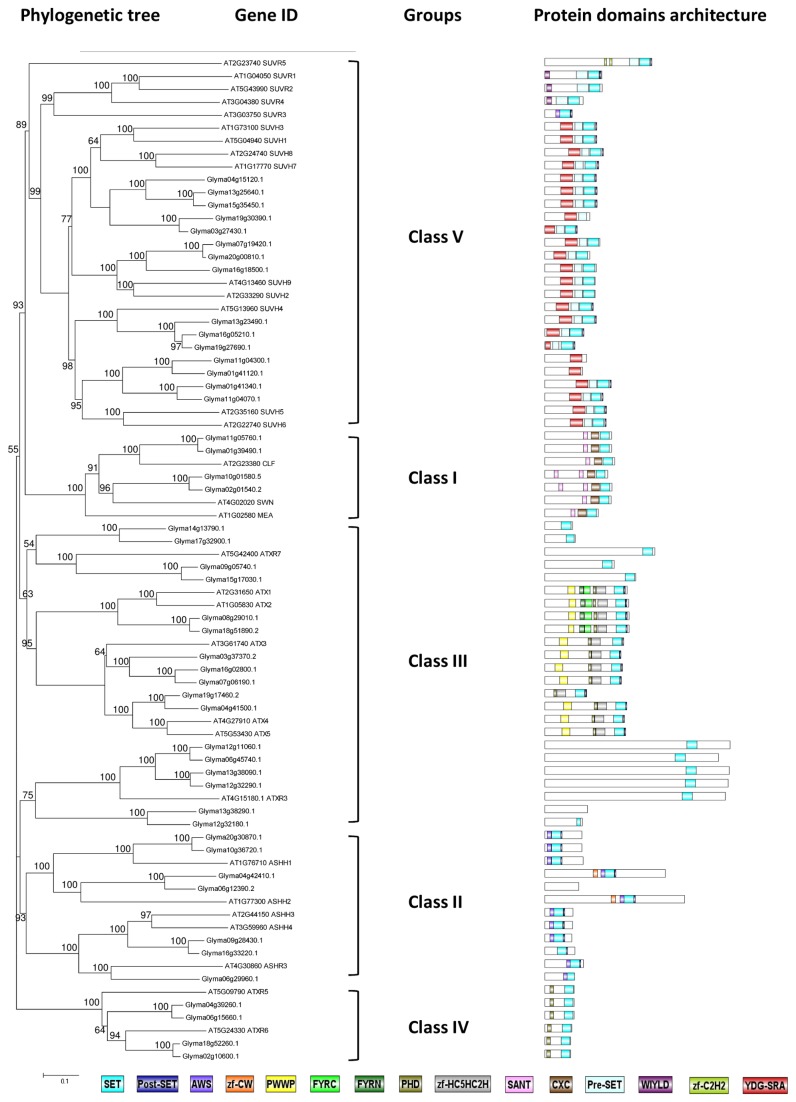
Phylogenetic tree and domain architecture of histone methyltransferase (HMTs). Neighbour-Joining phylogenetic tree are constructed based on protein alignments of *Arabidopsis* and soybean HATs proteins using ClustalX2. Bootstrap values greater than 50% are shown at the nodes and tree is drawn to given scale of branch lengths. The schematic diagrams show the domain organization of these proteins according to analysis by NCBI Batch-CD, SMART and PFAM searches. Different domains are indicated by the use of different colours as shown at the bottom of the figure. The accession numbers for the sequences used in the alignment are listed in the figure.

**Figure 8:**
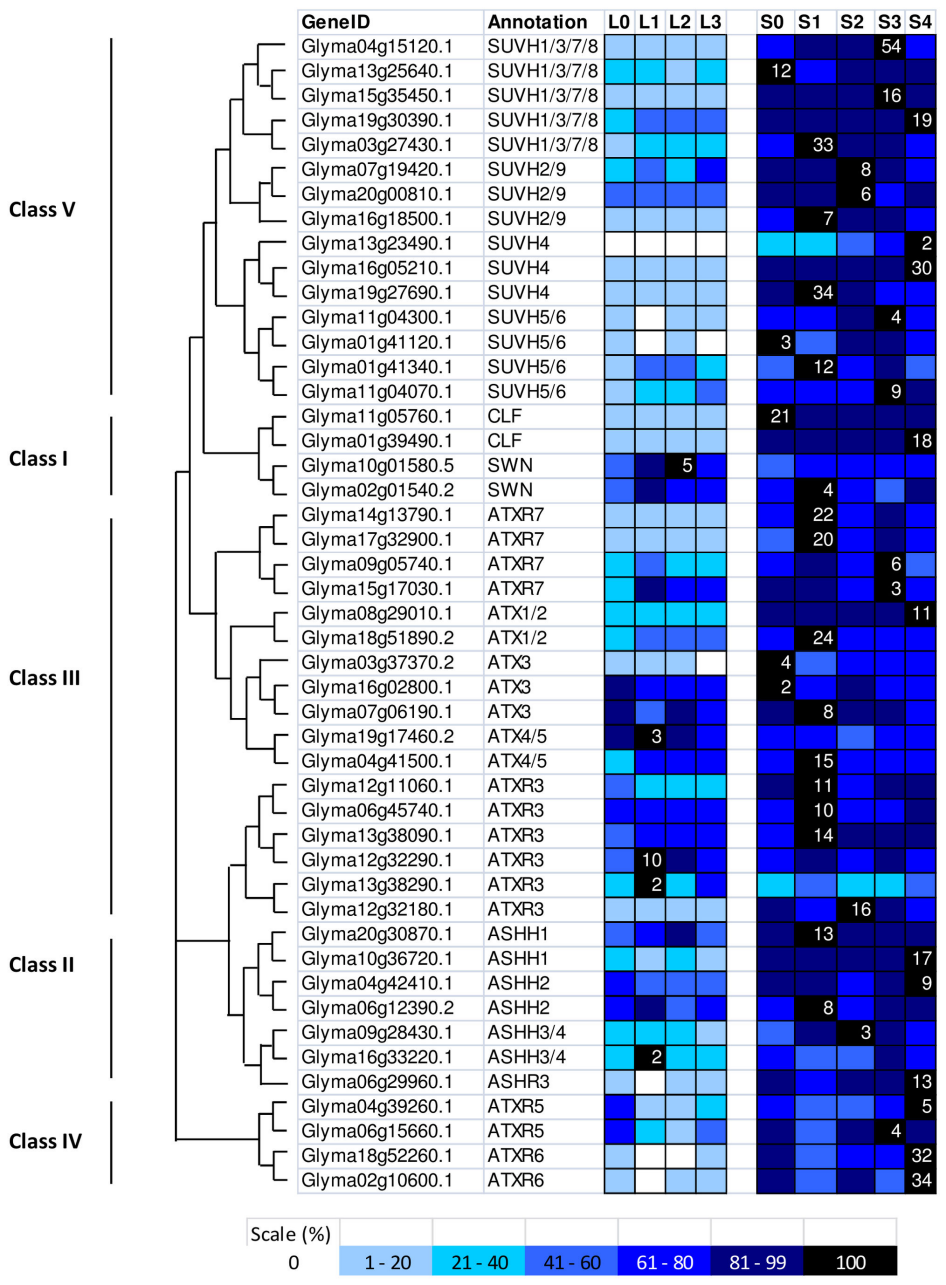
Expression profile and evolutional pattern of histone methyltransferase (HMTs). The heat map shows the relative abundances of soybean genes identified. The highest expression level for each gene across different samples is given in RPKM value. The level of expression for a gene across different samples are represented as percentage of the maximum expression level in colour code from 0% (white) to 100% (black). Heat maps were plotted against the phylogenetic tree constructed using soybean protein sequences and GO annotation. L0, L1, L2, and L3 were leaves at 0 short-day, 1 short-day, 2 short-day, and 3 short-day; S0, S1, S2, S3, and S4 were shoot apical meristems at 0 short-day, 1 short-day, 2 short-day, 3 short-day and 4 short-day.

The 47 SDG proteins in soybeans belong to five classes according to the protein sequence homology with *Arabidopsis* SDGs (Figure 7). Specifically, 4 soybean proteins are homologous to Class I SDGs (CLF, SWN, and MEA) with a similar domain architecture involving SANT, CXC, and SET domains. Two soybean proteins were homologous to each of the *Arabidopsis* Class I SDGs, CLF and SWN, but distinct expression patterns spotted between the two paralogues (Figure 8) and homologues of *MEA* were not found. In *Arabidopsis*, Class I SDGs work specifically as H3K27 methyltransferases and have been shown to directly induce the expression of *FT* and *FLC* in order to regulate flowering time [62]. Seven soybean proteins belong to the Class II SDGs, all showing a domain arrangement similar to that of the five *Arabidopsis* members, including the AWS (subdomain of Pre-SET), SET and Post-SET domains. All of the seven genes show a higher expression level in the SAM than in the leaf after an inductive short-day treatment except for one homologue (Glyma16g33220.1) encode for soybean ASHH3/4 (Figure 8). In *Arabidopsis*, ASHH2 is a H3K36 methyltransferase and is required for plant reproduction, especially in ovule and anther development [63]. 

Based on the domain organization, Class III is a diverse group that contain domains such as PWWP (with Pro-Trp-Trp-Pro motif for protein interaction), FYRC, FYRN (found in chromatin-associated proteins), PHD (plant homeodomain), zf-HC5HC2H (zinc finger) in addition to SET and Post-SET domains. There are 17 soybean proteins cluster with 7 *Arabidopsis* Class III SDGs (Figure 7). In *Arabidopsis*, Class III proteins - ATXR3, ATX1, and ATX2, are known to methylate H3K4 while ATXR7 in the separate clade is capable of methylating H3K36 (Figure 1) [36,64]. ATX1, ATX2 together with ATXR7 are responsible for preventing flowering happen before vernalisation through activation of floral repressor, *FLC* expression [36]. As soybean does not have a vernalisation requirement for flowering, it is interesting to identify the roles of the soybean Class III proteins in flowering or in SAM organogenesis, as most of them show high expression levels in the soybean SAM (Figure 8). In *Arabidopsis*, the enrichment of specific histone marks - H3K56ac and H3K4me3 were found on the core clock components, *CCA1* (*CIRCADIAN CLOCK ASSOCIATED 1*), *LHY* (*LATE ELONGATED HYPOCOTYL*) and *TOC1* (*TIMING OF CAB EXPRESSION 1*) and both histone marks display oscillating rhythms which coincide with the transcript patterns of the clock genes [65–67]. Study has showed that ATXR3 might responsible for H3K4me3 marks at these core clock genes [67]. Our phylogenetic analysis further supports the presence of Class IV SDGs (ATXR5 and ATXR6) which are only discovered in land plants. ATXR5 and ATXR6 in *Arabidopsis* are H3K27 monomethyltransferase while in mammals ENHANCER OF ZESTE HOMOLOG (EZH 1 and EZH2) function redundantly to control H3K27me1 mark [37]. H3K27me1 together with H3K9me2 are the two well-known repression marks associated with heterochromatin formation and gene silencing in both animals and plants. Interestingly, their four soybean homologues showed increased transcript levels after short-day treatment and peaked at 3 or 4 short days. 

The Class V SDGs is the largest group of SDGs protein in plants including fourteen *Arabidopsis* members separated into two main clades (Figure 7). The first subgroup consists of SUVR1-5 containing Pre-SET/AWS, SET, and Post-SET domains and SUVR1, SUVR2, and SUVR4 carry extra N-terminal WIYLD domains. The WIYLD domain of SUVR4 has been shown in *Arabidopsis* to bind to ubiquitin and enable SUVR4 to specifically trimethylate H3K9 (H3K9me3) to defence against detrimental transposon activity [68]. Intriguingly, no soybean homologue was being identified as members of this subgroup in the Class V SDGs from the transcriptome data of leaf and SAM during soybean flower initiation. The second subgroup is made up of nine *Arabidopsis* proteins – SUVH 1-9 which all contain an additional YDG-SRA domain. YDG-SRA is the abbreviation for SET and Ring finger Associated domain (SRA) with YDG motif which only present in chromatin-associated ubiquitin ligase in animals and Class V SDGs in plants [69]. Fifteen soybean proteins are classified into these subgroups and all showed higher expression levels in the SAM compared to the leaf (Figure 8). There are five homologues for SUVH1, 3, 7, and 8; three for SUVH2 and 9; three for SUVH4, and four for SUVH5, 6.

#### Soybean PRMTs

**Figure 9:**
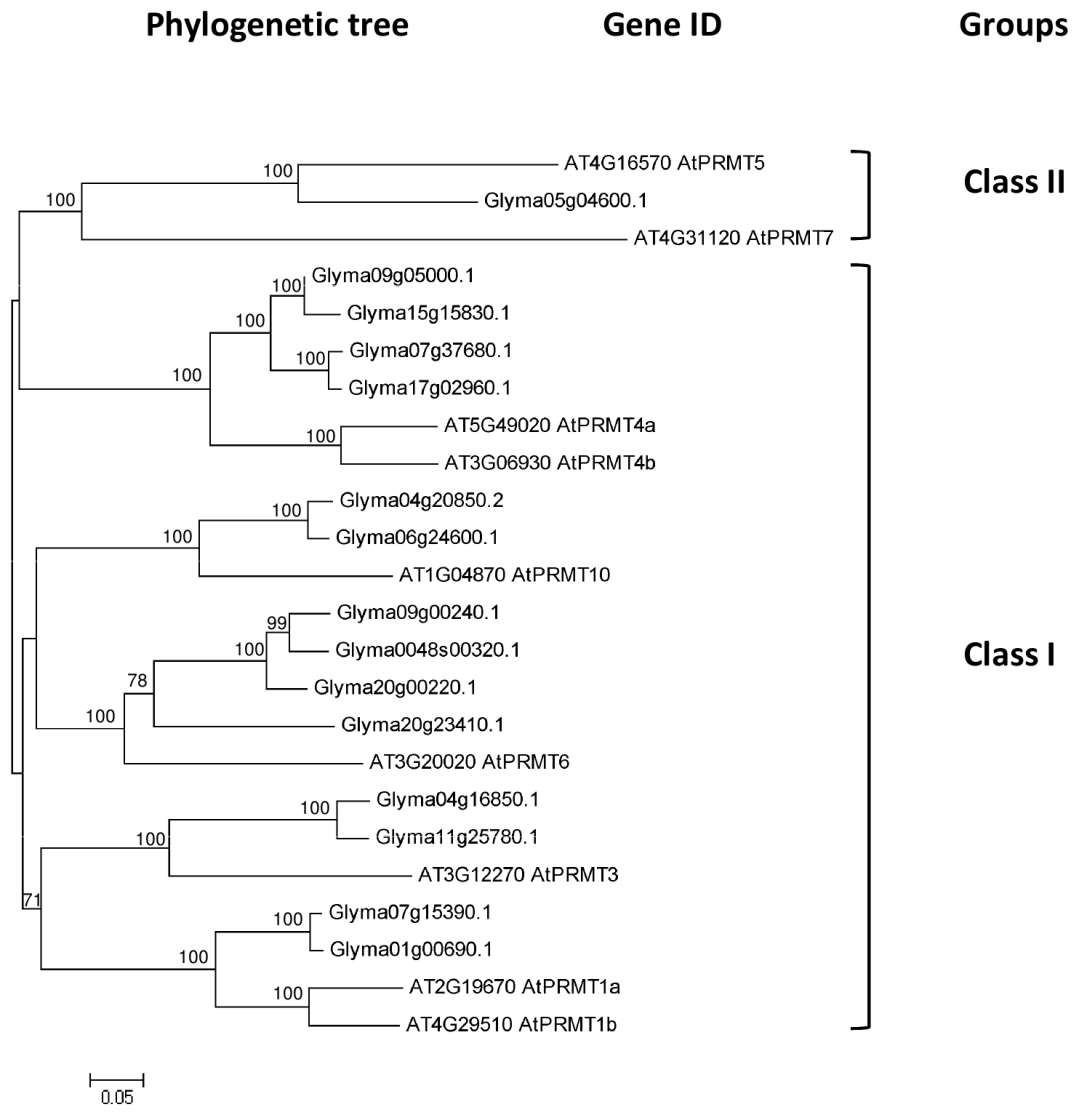
Phylogenetic tree and domain architecture of protein arginine methyltransferase (PRMTs). Neighbour-Joining phylogenetic tree are constructed based on protein alignments of *Arabidopsis* and soybean HATs proteins using ClustalX2. Bootstrap values greater than 50% are shown at the nodes and tree is drawn to given scale of branch lengths. The schematic diagrams show the domain organization of these proteins according to analysis by NCBI Batch-CD, SMART and PFAM searches. Different domains are indicated by the use of different colours as shown at the bottom of the figure. The accession numbers for the sequences used in the alignment are listed in the figure.

**Figure 10:**
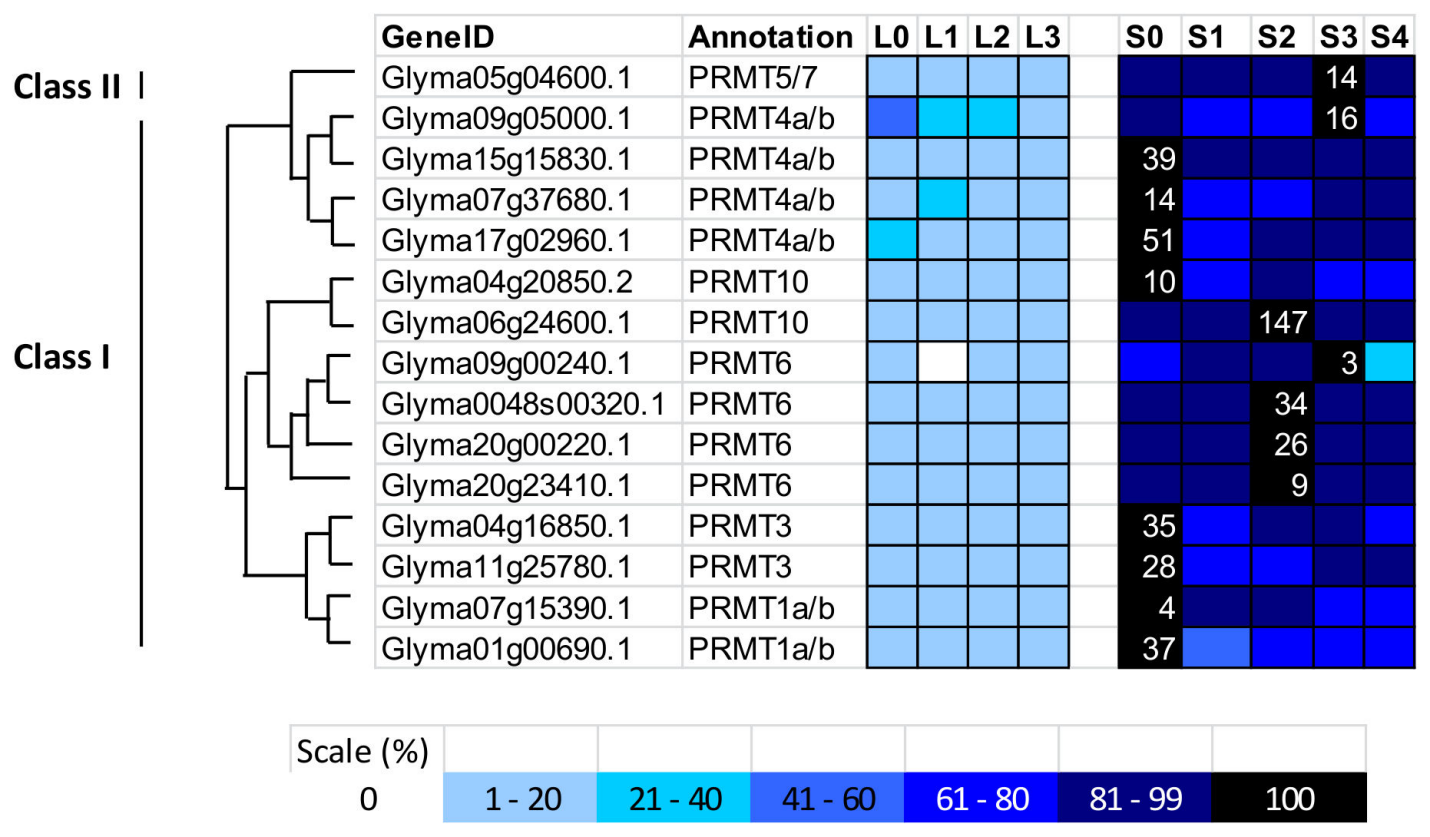
Expression profile and evolutional pattern of protein arginine methyltransferase (PRMTs). The heat map shows the relative abundances of soybean genes identified. The highest expression level for each gene across different samples is given in RPKM value. The level of expression for a gene across different samples are represented as percentage of the maximum expression level in colour code from 0% (white) to 100% (black). Heat maps were plotted against the phylogenetic tree constructed using soybean protein sequences and GO annotation. L0, L1, L2, and L3 were leaves at 0 short-day, 1 short-day, 2 short-day, and 3 short-day; S0, S1, S2, S3, and S4 were shoot apical meristems at 0 short-day, 1 short-day, 2 short-day, 3 short-day and 4 short-day.

As for methylation on arginine residue, there are 15 PRMTs in soybean when compared to 9 in *Arabidopsis* (Figure 9). There are two classes of PRMTs in plants – Class I will result in asymmetric dimethylarginine and Class II results in symmetric dimethylarginine while both are capable of catalysing formation of monomethylarginine [39]. From the phylogenetic analysis, 14 PRMTs are categorised as Class I PRMTs whereas 1 are Class II PRMTs (Figure 9). In higher plants such as *Arabidopsis* and rice, PRMTs were found to be involved in transcription and RNA processing [70]. AtPRMT4a/b, AtPRMT5, AtPRMT10 have been reported to regulate *FLC* expression in response to vernalisation. The transcriptome data of current study showed that most of the soybean *PRMTs* are highly expressed in the SAM with 8 of them peak at 0-short-day (before short day treatment), 4 peaking at 2 short days and 3 peaking at 3-short-day (Figure 10). Among them, Glyma06g24600.1, a homologue to *AtPRM10* has an extremely high expression level after two successive inductive short days. A mutation in *AtPRMT10* will result in the upregulation of *FLC* and a late-flowering phenotype, suggesting its function in promoting flowering in *Arabidopsis* [71]. However, the double mutant *atprmt5 atprmt10* showed an additive effect on both the *FLC* level and the delay of flowering time, implicating the involvement of *AtPRMT10* in other flowering pathways. As a plant not dependent on vernalisation for flowering, it will be interesting to study the *PRMT10* function in soybean. 

#### Soybean Jmjs

**Figure 11:**
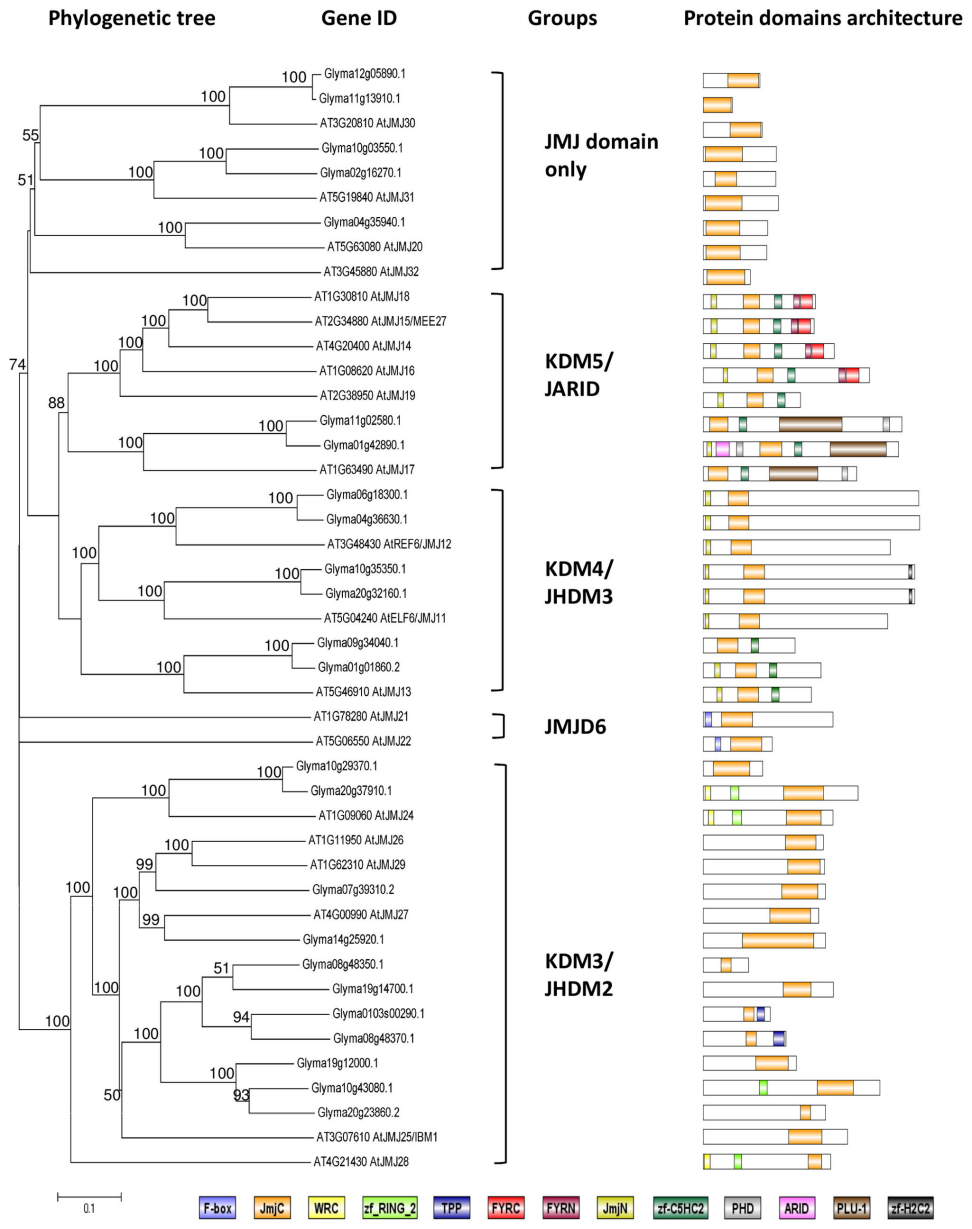
Phylogenetic tree and domain architecture of Jumonji proteins (JmjCs). Neighbour-Joining phylogenetic tree are constructed based on protein alignments of *Arabidopsis* and soybean HATs proteins using ClustalX2. Bootstrap values greater than 50% are shown at the nodes and tree is drawn to given scale of branch lengths. The schematic diagrams show the domain organization of these proteins according to analysis by NCBI Batch-CD, SMART and PFAM searches. Different domains are indicated by the use of different colours as shown at the bottom of the figure. The accession numbers for the sequences used in the alignment are listed in the figure.

The 21 JmjC-containing proteins in *Arabidopsis* can be classified into five classes [42,72]. In this study, there are 24 JmjC proteins found in soybean, but there is no homologue identified for a few of the JmjC proteins of KDM5 and JMJD6 classes such as AtJMJ14, AtJMJ15, AtJMJ16, AtJMJ18, AtJMJ19, AtJMJ21, AtJMJ22, AtJMJ28, and AtJMJ32 in the soybean transcriptome data during flower initiation (Figure 11). In the KDM5/JARID class, only two soybean homologues were found. The class is divided into two main clades: the first includes protein with C-terminal FYRN and FYRC domains, which are found in a variety of chromatin-associated proteins and the second includes protein with a PLU-1 domain, which play a role in DNA-binding. However only 2 soybean homologues cluster with AtJMJ17 in the second clade, no soybean homologue was found for first clade. On the other hand, there are 11 JmjC-containing soybean proteins in the class of KDM3/JHDM2 but 7 of them form a separate clade than those found in *Arabidopsis*. Interestingly, there are two proteins in the KDM3/JHDM2 class carry thiamin pyrophosphate (TPP) domains and other four contain zf_RING_2 domains. This domain combination suggested potential connection of histone demethylation with oxidative stress tolerance and ubiquitination activity [73]. The KDM4/JHDM3 class in plants consist of AtREF6/AtJMJ12, AtELF6/AtJMJ11, and AtJMJ13. Two soybean homologues were found in soybean transcriptome for *AtREF6* and *AtELF6* respectively and five soybean proteins for the close homologues of *AtJMJ13*. Besides JmjC domains, the two soybean homologues of AtELF6 also carry C-terminal zinc finger H2C2 domain (zf-H2C2). The JMJD6 proteins include AtJMJ21 and AtJMJ22 but no clear soybean homologue was found in this study. The JMJD6 demethylase are known to be arginine demethylase in animals [44]. In the phylogenetic tree (Figure 11), a clade of JmjC domain only proteins were created, containing soybean homologues for AtJMJ30, AtJMJ31, AtJMJ21 and without clear soybean homologues for AtJMJ32.

**Figure 12:**
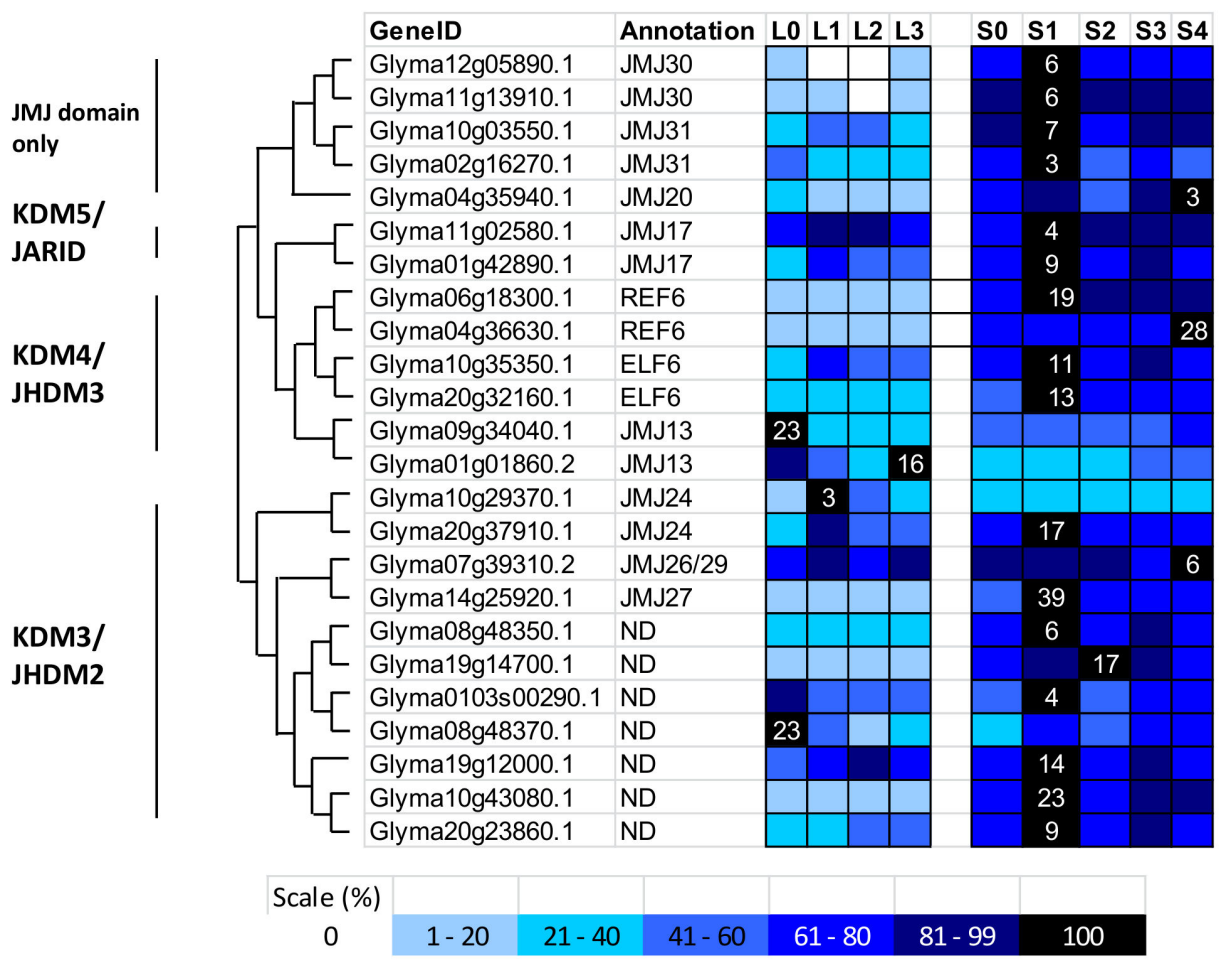
Expression profile and evolutional pattern of Jumonji proteins (JmjCs). The heat map shows the relative abundances of soybean genes identified. The highest expression level for each gene across different samples is given in RPKM value. The level of expression for a gene across different samples are represented as percentage of the maximum expression level in colour code from 0% (white) to 100% (black). Heat maps were plotted against the phylogenetic tree constructed using soybean protein sequences and GO annotation. L0, L1, L2, and L3 were leaves at 0 short-day, 1 short-day, 2 short-day, and 3 short-day; S0, S1, S2, S3, and S4 were shoot apical meristems at 0 short-day, 1 short-day, 2 short-day, 3 short-day and 4 short-day.

 Interestingly, most of the soybean JmjC proteins (Figure 12) show transcript abundance in the SAM after exposure to 1 short day, suggesting the involvement of JmjC protein-mediated demethylation and potential gene activation in the SAM after exposure to the inductive short-day. AtJMJ14 and AtELF6 are H3K4 demethylases and have recently been shown to act together to demethylate the H3K4me3 mark of the floral stimulus, *FLOWERING LOCUS T* (*FT*) locus, repressing *FT* expression [74,75]. The *FT* locus is also enriched with the H3K27me3 mark, and LIKE-HETEROCHROMATIN PROTEIN 1 (LHP1) is an effector protein that binds to H3K27me3 and reduces its expression [76]. A recent research paper has demonstrated that AtREF6 (RELATIVE OF ELF6) is a H3K27 demethylase [77], suggesting that AtREF6 might work together with LHP1 to regulate the H3K27me3 marks. As mentioned before, CLF and SWN are H3K27 methyltransferases and directly regulate the expression of *FT* and *FLC* in order to regulate flowering time. Together, these results suggest that AtREF6 might act antagonistically to CLF and SWN to regulate *FT* expression at the epigenetic level. The findings of this study further support this hypothesis, as the soybean paralogues of *REF6* showed an expression profile opposite to that of *CLF* paralogues (Figure 8, 12). The other members of the KDM4/JHDM3 class, JMJ13, has two soybean homologues, showed a higher transcript level in the leaf than in the SAM, and opposing expression profiles were found between the two paralogues, where one was highly expressed before the short-day treatment and the other peaked after 3 short days. In *Arabidopsis*, *JMJ30* is involved in circadian clock regulation and is the only JmjC protein in *Arabidopsis* that shows an oscillation pattern with circadian rhythms [78]. In plants, the circadian clock plays a vital role in the flowering pathway, especially in the photoperiodic control of flowering time. The transcriptome profile for soybean *JMJ30* homologues peaks at 1 short day in the SAM, suggesting that *JMJ30* might be involved in the circadian clock regulation of photoperiodic flowering in soybean. 

### Transcript profile of histone modifiers in soybean during flowering initiation

**Figure 13:**
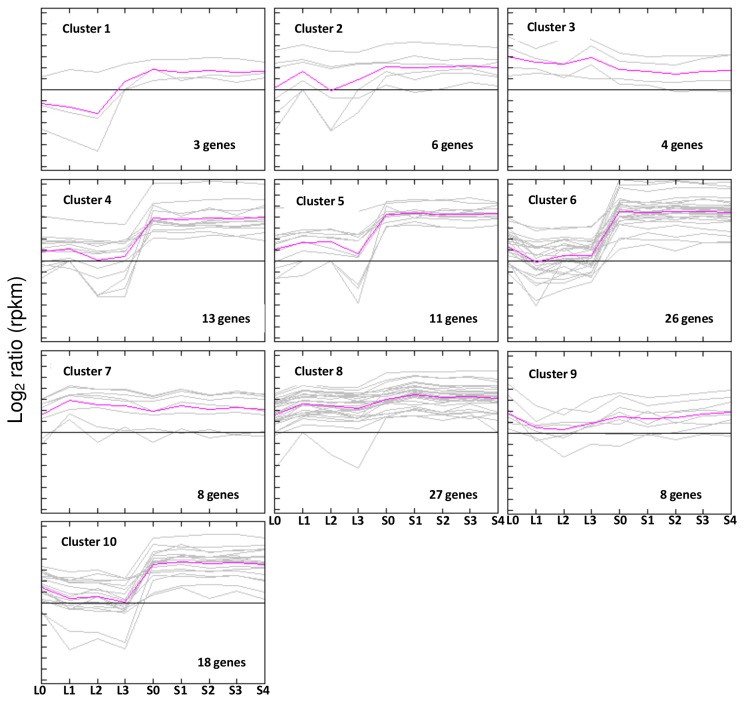
Expression cluster analysis of 152 histone modifiers genes in soybean. The samples included are 152 soybean histone modifiers genes identified in this study and their expression ratio (log_2_ RPKM) from nine samples during flowering initiation process. K-means clustering was performed with Person correlation algorithm and number of clusters = 10 and number of iterations = 50. The grey line show expression patterns of each gene and the purple line represent the average expression profile of particular cluster (cluster 1 - 10). L0, L1, L2, and L3 were leaves at 0 short-day, 1 short-day, 2 short-day, and 3 short-day; S0, S1, S2, S3, and S4 were shoot apical meristems at 0 short-day, 1 short-day, 2 short-day, 3 short-day and 4 short-day.

In order to gain insight into overall expression profiles of histone modifiers in soybean during flowering initiation process, a K-mean clustering analysis was performed based on a Pearson correlation showing 10 clusters for the *in silico* expression data of 124 histone modifiers genes (Figure 13). List of genes in the 10 clusters was included in Table S2.

 Of the 124 histone modifiers genes identified in current study, only 12 showed higher level of expression in the leaf than in the SAM. These 12 genes are grouped in Cluster 3 and 7 (Figure 12). The genes in Cluster 3 include soybean homologue for *HDA14*, one of the two homologues of *SRT2*, *JMJ13*, and *ATX4*/*5* (Figure 6, Table S2). Genes in Cluster 7 are mostly genes involved in histone acetylation or deacetylation.

Additionally, most of the histone modifiers genes (104 genes) have higher expression at SAM when compared to leaf and fell into Cluster 1, 2, 4, 5, 6, 8, and 10 (Figure 13). Among these clusters, genes from Cluster 4, 5, 6, and 10 are highly expressed in the SAM tissues. The genes in different clusters had slightly different transcript profiles in different stages of short-day treatment, such as genes of Cluster 4 has an increased expression in the leaf at 1-short-day, and Cluster 5 show a decreased expression in the leaf at 3-short-day. Cluster 6 is one of the largest cluster among the 10 clusters, containing 26 genes which show highest expression in the SAM especially 2-4 short-day, Cluster 10 showed higher expression before inductive short-day at both leaf and SAM tissues (L0 and S0), Cluster 1, 2, and 8 still showed higher expression level at SAM than leaf but the difference is small, such as Cluster 1 has extremely low expression in leaf and low expression in SAM, Cluster 2 has overall low expression in both tissue types but show lowest expression level at leaf after 2 short days (L2), and Cluster 8 shows an increase in expression levels after 1 short day in both the leaf and SAM (L1 and S1).

There are 8 genes of Cluster 9 of which have overall low transcript level at both leaf and SAM tissue of different stages including soybean homologues for HMTs such as *ATX3*, *ATXR5*, *SRT2*, and *SUVH4* (Figure 13, Table S2). 

### RNAi silencing

**Figure 14:**
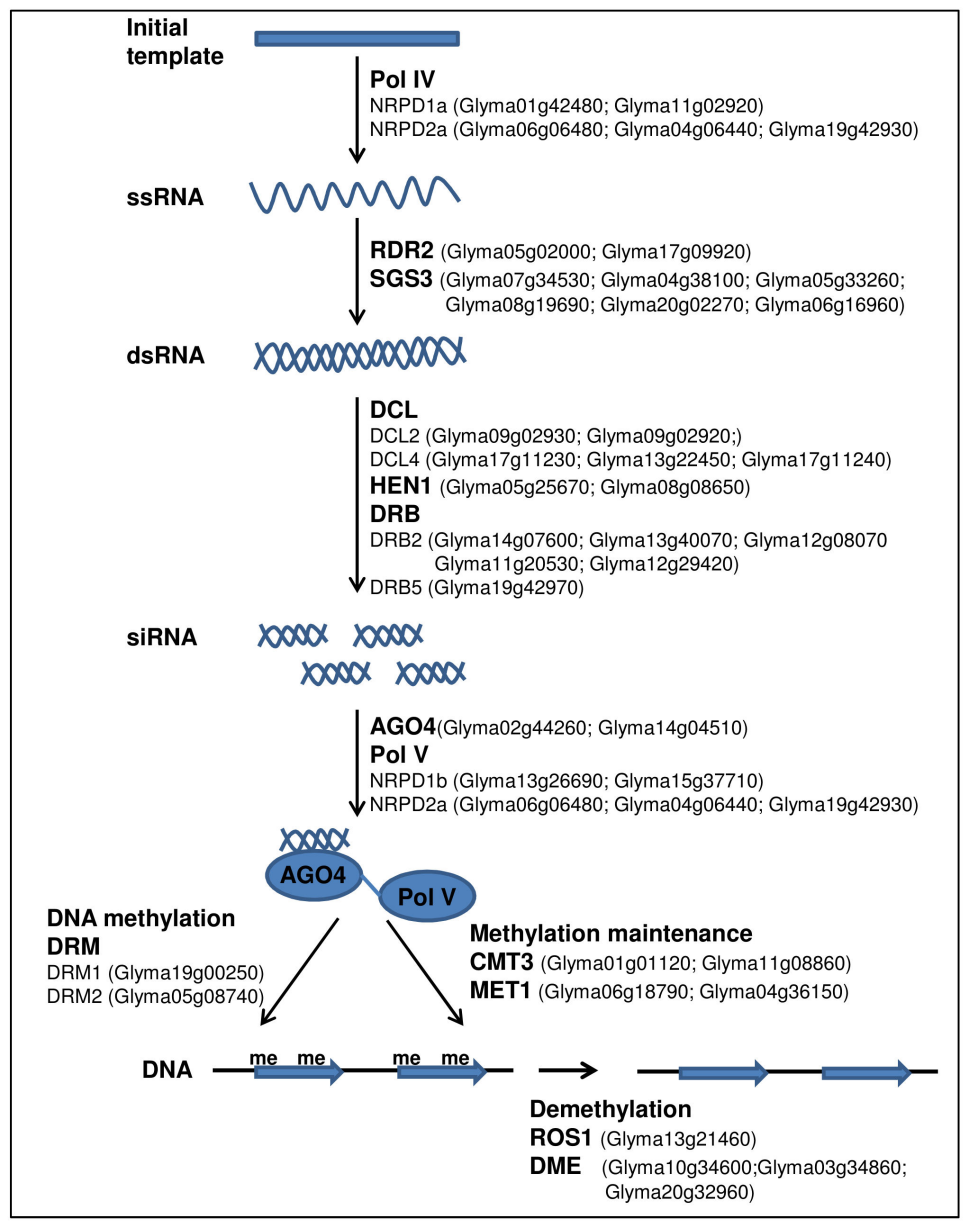
An outline of RNA-mediated chromatin silencing pathway showing soybean homologues for *Arabidopsis* RNAi genes. Pol IV (RNA polymerase IV), RDR 2 (RNA-dependent RNA polymerase 2), SGS3 (Suppressor of gene silencing), DCL (Dicer-like), HEN1, DRB (Double-strand RNA binding), AGO4 (Argonaute 4), Pol V (RNA polymerase V), DRM (Domains rearraged DNA methylation 2), CMT3 (Chromomethylae 3), MET1 (Methyltransferase 1), ROS1 (Repressor of silencing), DME (Demeter).

**Figure 15:**
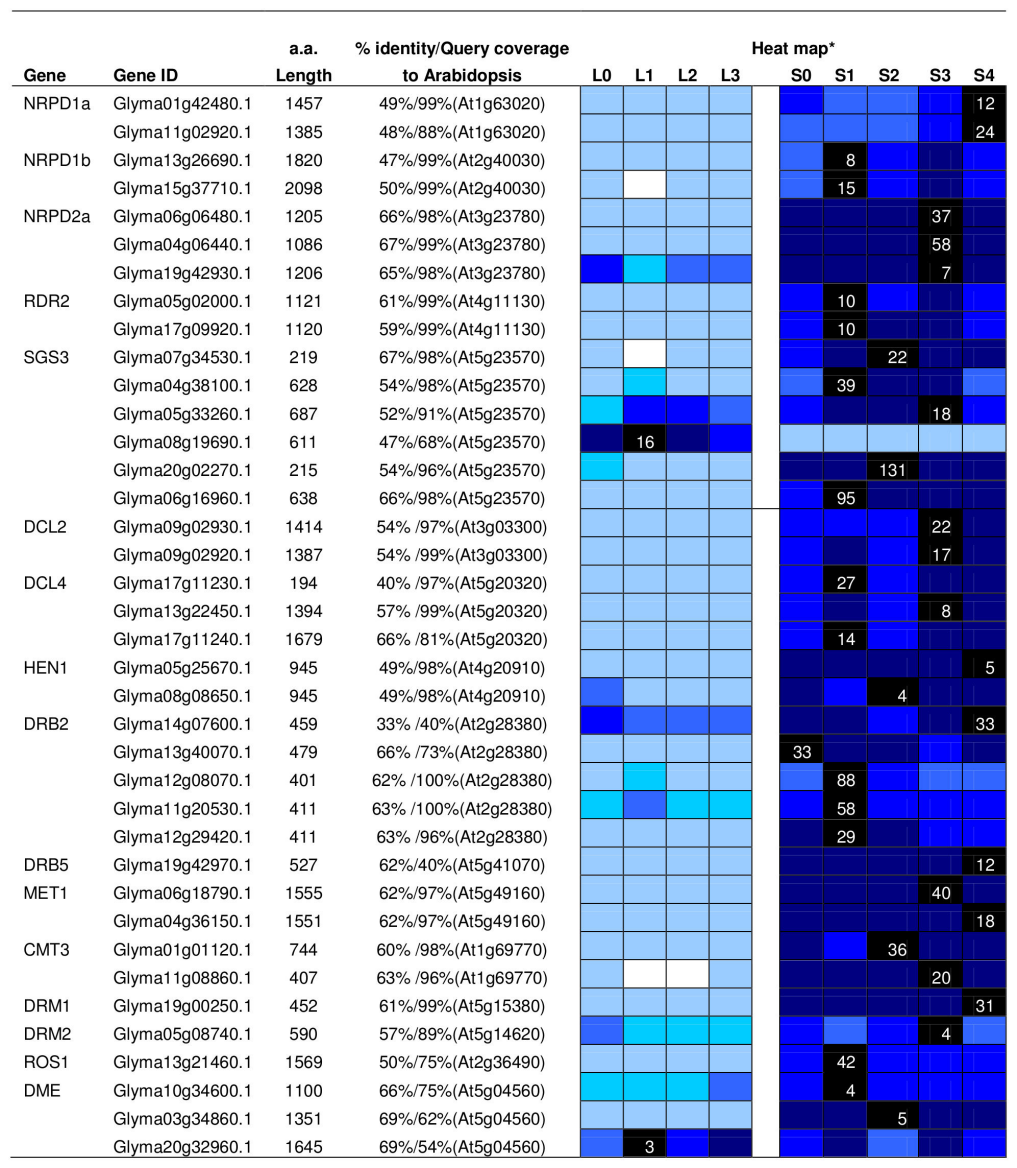
List of RNAi associated genes identified in soybean and their expression patterns during flowering initiation. The heat map shows the relative abundances of soybean genes identified. The highest expression level for each gene across different samples is given in RPKM value. The level of expression for a gene across different samples are represented as percentage of the maximum expression level in colour code from 0% (white) to 100% (black). L0, L1, L2, and L3 were leaves at 0 short-day, 1 short-day, 2 short-day, and 3 short-day; S0, S1, S2, S3, and S4 were shoot apical meristems at 0 short-day, 1 short-day, 2 short-day, 3 short-day and 4 short-day.

There is emerging evidence that ncRNAs are associated with epigenetic mechanism to regulate gene transcription via RNA interference silencing [79,80]. Here we not only identified 47 soybean genes that might play a role in RNAi and associated pathways (Figure 14), but also provided their expression profile during flowering initiation (Figure 15). Figure 15 shows the putative soybean homologues of RNAi associated genes. A high percentage of similarity in protein sequence was found between soybean and its *Arabidopsis* counterpart with the lowest value being 33%. 

 In plants, RNA interference silencing is generally referred to RNA-directed DNA methylation (RdDM), where siRNAs are synthesised and followed by *de novo* methylation [81,82]. The core components of the machinery are RNA polymerase, double-strand RNA binding (DRB), dicer-like (DCL), argonaute (AGO), DNA methyltransferase, and DNA glycosylase. Figure 14 showed the simplified diagram for RdDM pathway which contains putative soybean orthologs of *Arabidopsis* RNAi genes.

 The RNA polymerases involve in *de novo* methylation are RNA polymerase IV (NRPD1a and NRPD2a) and V (NRPD1b and NRPD2a) where RNA polymerase IV responsible for synthesis of ssRNA, RNA polymerase V works together with AGO4-bounded siRNAs to target cytosine methylation [83,84]. Two soybean homologues for *NRPD1a*, largest subunit of RNA polymerase IV, showed increased expression after exposure to short-day whereas two soybean homologues for *NRPD1b*, largest subunit of RNA polymerase V, showed a peak at 1-short-day followed by gradually reduced expression (Figure 15). NPRD2a which is the second largest subunit for both RNA polymerase are constantly highly expressed at the SAM for the three soybean homologues found. RDR2 together with SGS3 (SUPPRESSOR OF GENE SILENCING) use the ssRNA as a template to synthesise dsRNA. Two soybean homologues were found for *RDR2* and they have an expression pattern similar to that of *NPRD1b* while six soybean *SGS3* homologues were found with one shows higher expression in leaf than SAM.

 Followed by dsRNA synthesis, DCL and HEN1 come into play and process dsRNA into siRNAs in a DRB associated complex [85,86]. *Arabidopsis* genome encodes five DCL and six DRB proteins and they were found to interact in vivo in the siRNA pathway [87]. Five *DCL* genes were identified in the transcriptome study - 2 homologues for *DCL2*, and 3 for *DCL4*. They all show higher expression level at SAM after short-day treatment. Two soybean *HEN1* homologues were identified and shown to have higher expression at SAM. Six *DRB* homologues were found in soybean with five homologous to *DRB2* and 1 for *DRB5*. The expressions of them vary between paralogues, with with some peaking at 1 short day and some peaking at 4 short days (Figure 15).

**Figure 16:**
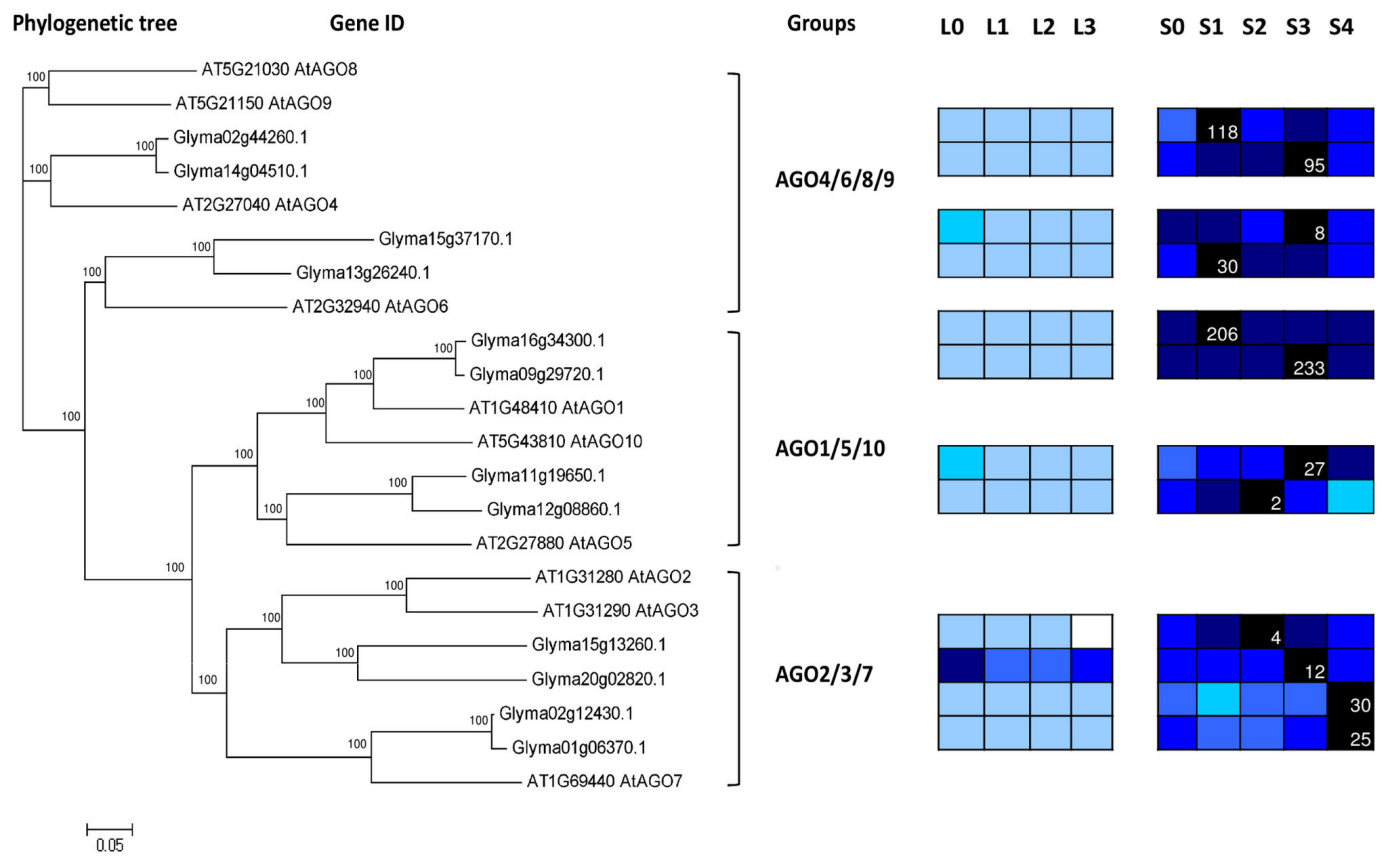
Phylogenetic tree of Argonaute proteins (AGO). Neighbour-Joining phylogenetic tree are constructed based on protein alignments of *Arabidopsis* and soybean HATs proteins using ClustalX2. Bootstrap values greater than 50% are shown at the nodes and tree is drawn to given scale of branch lengths. Heat maps were plotted against the phylogenetic tree constructed using soybean protein sequences and GO annotation. L0, L1, L2, and L3 were leaves at 0 short-day, 1 short-day, 2 short-day, and 3 short-day; S0, S1, S2, S3, and S4 were shoot apical meristems at 0 short-day, 1 short-day, 2 short-day, 3 short-day and 4 short-day.

 A phylogenetic analysis of the AGO proteins groups the proteins into three main clades, with AtAGO4, AtAGO6, AtAGO8 and AtAGO9 in the first clade; AtAGO1, AtAGO5, AtAGO10 in the second clade; AtAGO2, AtAGO3, and AtAGO7 in the third clade (Figure 16). In this study, twelve soybean AGO genes were identified and shown to be homologues of *AtAGO1*-*10* [88]. Interestingly, the transcriptome data of soybean leaf and SAM during flowering initiation process showed higher expression level of *AGO* genes in SAM than leaf and they all peak after induction of short-day (Figure 16). *AGO1* has the highest expression value amongst the AGO genes. In *Arabidopsis*, *AtAGO1* was shown to be involved in miRNA pathway and regulate leaf polarity [89]. It is intriguing to learn that soybean *AGO1* paralogs are both have low transcript abundance in the leaf. *AGO10* is the closet member to *AGO1*, *AtAGO10* has been shown to have a role in SAM development in addition to organ polarity by repressing miRNA166/165 [90,91]. Indeed, miRNA166 and miRNA166* have been isolated in soybean, and the expression of miRNA166* was observed in the peripheral region of the SAM [92]. Surprisingly, no apparent homologue of *AGO10* was found in soybean leaf or SAM transcriptome. A possible explanation could be the role of *AGO10* in soybean is carried out by other *AGOs* such as the closely related *AGO1* genes. AGO on the first clade, AtAGO4, AtAGO6, AtAGO8 and AtAGO9 are the important AGO proteins involved in RNAi machinery which will bind to siRNAs to silence specific loci [93]. AtAGO7 is particularly important for trans-acting siRNA (ta-siRNA) production by recruiting miR390 and work predominantly to regulate transition from juvenile to adult vegetative phase [94]. The transcript profile of the soybean *AGO7* homologues showed gradually increased transcription level with progression of short-day period (Figure 16). 

 DRM1/2, MET1, CMT3 are all known as DNA methyltransferase involve in RNA-directed DNA methylation [95]. DRM1 and DRM2 are the proteins involved in *de novo* methylation which direct siRNAs for cytosine methylation in homologous regions on specific loci [96]. One soybean homologue was found for *DRM1* which is highly expressed at SAM at 4-short-day while single homologue was identified for *DRM2* which has highest expression at SAM at 3-short-day (Figure 15). On the other hand, MET1 and CMT3 are responsible for maintenance of CG and CHG methylation, respectively. Some of the HMTs such as SUVH4 and SUVH5 also involve in locus specific methylation maintenance [97]. Two soybean homologues were identified for each of the *MET1* and *CMT3* genes and all four show high expression at the SAM. The *Arabidopsis* ROS1 and DME encode DNA glycosylases that are capable of demethylating methylcytosine from DNA [98]. One *ROS1* soybean homologue and three *DME* soybean homologues were recognised. One of the *DME* homologues (Glyma20g32960.1) showed a higher transcript level in the leaf than in the SAM (Figure 15). 

## Conclusions

We present here the transcriptome data of soybean histone modifiers and RNAi genes from nine different samples after an inductive short-day treatment to gain insight into the epigenetic mechanisms involve in soybean flowering initiation. By mining the transcriptome dataset, we identified *in silico* 124 histone modifier genes and 47 RNAi-associated genes in soybean. The 124 histone modifier genes include 14 histone acetyltransferases (HATs), 24 histone deacetylases (HDAs), 47 histone methyltransferases (HMTs), 15 protein arginine methyltransferases, and 24 JmjC domain-containing demethylases. The soybean histone modifiers were further characterised based on phylogenetic analysis and protein domain identification, reporting their classification regarding protein sequence homology and the possibility of a new class or domain combination in a particular protein family. By analysing the transcriptome data of all the histone modifier genes identified in current study, most of these genes were shown to have higher expression levels in the SAM than in the leaf. These results may suggest a higher rate of epigenetic modification of the SAM activities during the soybean flowering initiation process. Besides histone modifications, RNA-directed DNA methylation is another important mechanism for the epigenetic regulation. A total of 47 RNAi-associated genes in soybean were identified in this study, providing an overview of their expression during flowering initiation. The information gathered in the current study will be of particular interest for future studies on the divergence of functionality and the evolution of epigenetic-associated genes in flowering plants.

## Supporting Information

Table S1 **The raw transcriptome data of soybean histone modifiers and RNA silencing transcripts.**
(XLSX)

Table S2 **List of genes in 10 clusters of K-mean clustering analysis.**
(XLSX)
